# Enhanced YOLOv11n: A Method for Potato Peel Damage Detection

**DOI:** 10.1002/fsn3.70576

**Published:** 2025-07-04

**Authors:** Qiying Li, Ke Chen, Qian Wang, Fuxiang Wang, Weigang Deng

**Affiliations:** ^1^ College of Mechanical and Electrical Engineering Inner Mongolia Agricultural University Hohhot China; ^2^ Inner Mongolia Engineering Research Center of Intelligent Equipment for the Entire Process of Forage and Feed Production Hohhot China

**Keywords:** AFPN, EIEStem, MLCA, potato peeling, Re‐Calibration FPN, YOLOv11

## Abstract

Potatoes are susceptible to skin damage during harvest and transportation, which impacts storage capabilities, increases the risk of microbial contamination and decay, and diminishes both quality and food safety. Rapid detection of potato peel damage is crucial for the subsequent automatic sorting process. To address this, this paper introduces an enhanced YOLOv11n target detection algorithm for identifying damaged potatoes. The primary enhancements include: integrating the MLCA attention mechanism into the C3k2 module of the Backbone network to combine channel and spatial attention, thereby enhancing the network's ability to detect skin damage features; proposing a Re‐Calibration FPN to refine the Neck component, optimizing multi‐scale feature fusion through bidirectional fusion and an adaptive attention mechanism to accurately define the area of skin damage and improve the effectiveness of multi‐scale feature fusion; replacing the first two convolution layers of the Backbone with the EIEStem module, and combining SobelConv with convolutional branches to strengthen the feature representation of potato peel damage images; optimizing the detection head with AFPN to narrow the semantic gap, enhance feature fusion, and further improve the detection of potato peel damage. The WloU loss function is introduced to enhance the precision of boundary regression and reduce the occurrence of missed and false detections of skin damage. Comparative and ablation experiments demonstrate that the improved algorithm outperforms the benchmark YOLOv11n model by 6.9%, 15%, 7.4%, and 11.8% in terms of mAP@0.5, mAP@0.5:0.95, precision rate (P), and recall rate (R), respectively. Compared to existing mainstream algorithms, the proposed algorithm exhibits superior performance in comprehensive comparisons, fully validating its effectiveness in the task of potato peel damage detection.

## Graphical abstract

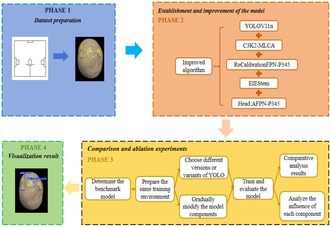

## 1 Introduction

Potatoes are the world's fourth largest food crop and among the top 10 nutritionally rich foods (Li et al. 2019). They are highly valued for their high yields, adaptability, comprehensive nutrition, and industrial applications (Bai et al. 2019). However, potatoes are prone to skin damage during harvesting and transportation, which reduces storage life and increases susceptibility to microbial invasion and rot, compromising quality and edibility (Furrer et al. 2017). Therefore, timely detection of potato surface damage is crucial for research and commercial applications.

At present, the detection of surface defects on Chinese potatoes predominantly relies on manual methods. These methods suffer from issues such as excessive labor intensity, low efficiency, significant subjectivity, high costs, and poor reliability, all of which impede the advancement of the processing automation industry (Su et al. 2018). However, with the advancement of computer technology, machine vision has been extensively applied in agriculture, and the use of image recognition to detect potato defects has emerged as a research focal point (Xu et al. 2017; Lv et al. 2019). Li et al. (2018) developed a potato bud eye recognition method using three‐dimensional geometric features of color saturation, achieving a recognition rate of 91.48% and an error rate of 4.32%. Wang (2021) proposed detection methods for various potato defects using the HSV color space for low brightness, RGB for green skin, and the SUSAN operator for complex lesions, with high accuracy in identifying defect areas. Liu et al. (2022) used grayscale, median filtering, and the Otsu method to segment potato images from backgrounds, achieving over 90% detection accuracy for worm holes, cracks, mechanical damage, rot, and green skin. Yu (2021) analyzed external potato defects using RGB Euclidean distance for green skin detection and the SUSAN operator for other defects. Previous studies mainly focused on detecting obvious defects in potatoes, such as insect eyes, mechanical damage, green skin, sprouting, and dry rot, which can be identified to some extent using conventional machine vision methods. However, broken skin areas in potatoes are less distinct from healthy areas, presenting greater detection challenges and fewer research efforts. Therefore, developing more precise and efficient technologies for identifying potato broken skin areas is especially important.

Deep learning, capable of automatically extracting high‐level features, is increasingly replacing traditional methods in agriculture due to its adaptability and robustness (Fang et al. 2019; Liang et al. 2019). YOLO, a single‐stage detector, outperforms two‐stage detectors like Faster R‐CNN in speed and accuracy (Ren et al. 2017), making it ideal for real‐time applications and widely used in potato surface defect detection. For example, Li et al. (2025) proposed an enhanced YOLO v5s model incorporating collaborative attention and ASPP modules, achieving high efficiency and accuracy for detecting green skin, mechanical damage, germination, and dry rot. Gu et al. (2019) developed an improved YOLO seed potato eye detection model using C3 Faster and GOLD‐YOLO structures, with significant gains in accuracy and parameter count. Liao et al. (2025) presented HCRP‐YOLO, based on YOLOv8n, integrating HGNetv2, CCFM, and RHead modules, and using Group Slimming pruning. It achieved a 1.1% increase in mAP and a 4.2% increase in recall compared to YOLOv8n. Zhang et al. (2021) enhanced YOLOv4 with a lightweight attention mechanism and Mobilenet V3, improving accuracy and speed for detecting normal and damaged potatoes. Yang, Lei, et al. (2023), Yang, Liu, et al. (2023) developed MDDNet, integrating Res2Net into YOLO v3‐tiny, achieving a detection accuracy of 90.26% and a speed of 75 ms per image.

This paper proposes an enhanced YOLOv11n algorithm for detecting potatoes with broken skin. The main improvements include: integrating the MLCA attention mechanism into the C3k2 module of the Backbone, introducing a Re‐Calibration FPN to refine the Neck, replacing the first two convolutional layers of the Backbone with the EIEStem module, applying AFPN to optimize the detection head, and incorporating the WloU loss function. These enhancements significantly improve detection efficiency and accuracy by enabling rapid identification and localization of broken skin areas, providing robust technical support for potato defect detection.

## 2 Materials and Methods

### 2.1 Model Construction

#### 2.1.1 Introduction and Improvement of YOLOv11 Algorithm

YOLOv11 was released by Ultralytics in September 2024 (Khanam and Hussain 2024). Its innovations include: replacing the traditional C2f with C3K2 in the backbone network and introducing the C2PSA module to enhance multi‐scale feature extraction; an improved spatial pyramid pool and rich feature expression from the C2PSA. The PAN‐FPN structure is used in the neck network to enhance target location capabilities. The detection head employs a decoupling design and DWConv operation to reduce model parameters and computational overhead. By reducing the number of network layers and parameters, YOLOv11n achieves faster inference speeds and a smaller model size, making it suitable for resource‐constrained devices. This paper uses this as the benchmark model.

Compared to YOLOv8 and YOLOv10, YOLOv11 exhibits significant enhancements in detection accuracy and speed. However, there remains potential for improvement in the detection of potato peel breaks. This paper proposes enhancements to YOLOv11, detailed as follows:

1. The MLCA attention mechanism is integrated into the C3k2 module within the backbone network to enhance its capability to capture potato peel features. By combining channel and spatial attention at both local and global levels, the mechanism effectively improves the model's accuracy in detecting potato peel.
2. A re‐calibration FPN was proposed to enhance the Neck component of the YOLOv11 network. Through the application of bidirectional fusion and an adaptive attention mechanism, the network can precisely delineate and recalibrate the potato peel region, thereby further enhancing the efficacy of multi‐scale feature fusion.
3. The first two convolutional layers in the Backbone section of the original network are replaced with EIEStem modules. These modules integrate the SobelConv branch for edge information extraction and the convolutional branch for spatial information extraction, thereby learning the feature representation of potato peel images more effectively.
4. Utilizing the Asymptotic Feature Pyramid Network (AFPN) to optimize the detection head of YOLOv11 assists in narrowing the semantic gap between features at various levels, enhancing the feature fusion effect. This allows the detection model to more effectively adapt to semantic information across different levels, thereby further enhancing the detection performance for potato peel defects;
5. The WloU loss function is introduced to address the issue that the traditional CIoU loss function overlooks the shape and scale of the target frame, thereby making border regression more accurate and reducing the occurrence of missing and false detections of broken skin.

**FIGURE 1:**
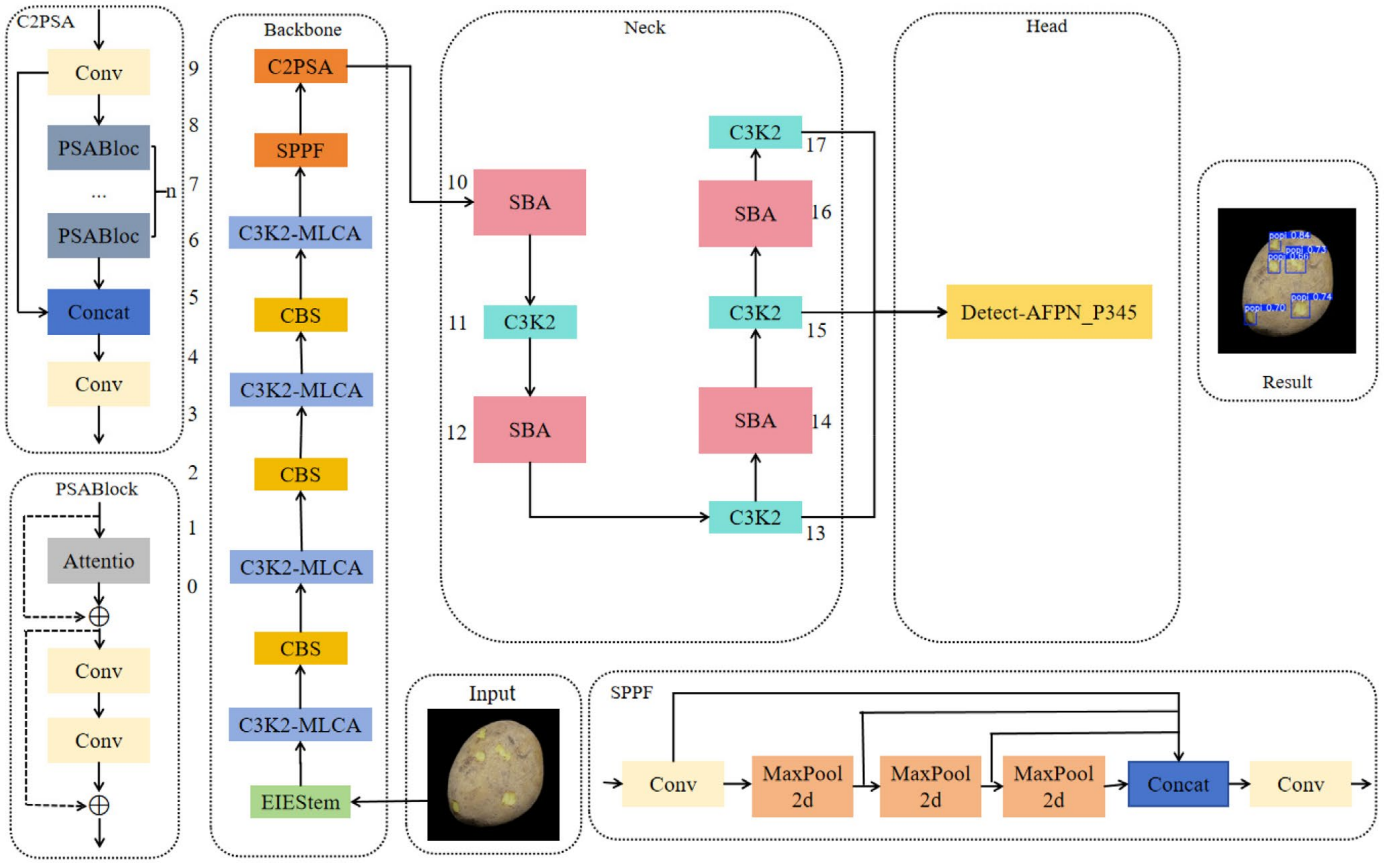
Improved network structure diagram.

The improved structure is shown in Figure 1.

#### 2.1.2 MLCA Mixes Local Channel Attention

**FIGURE 2:**
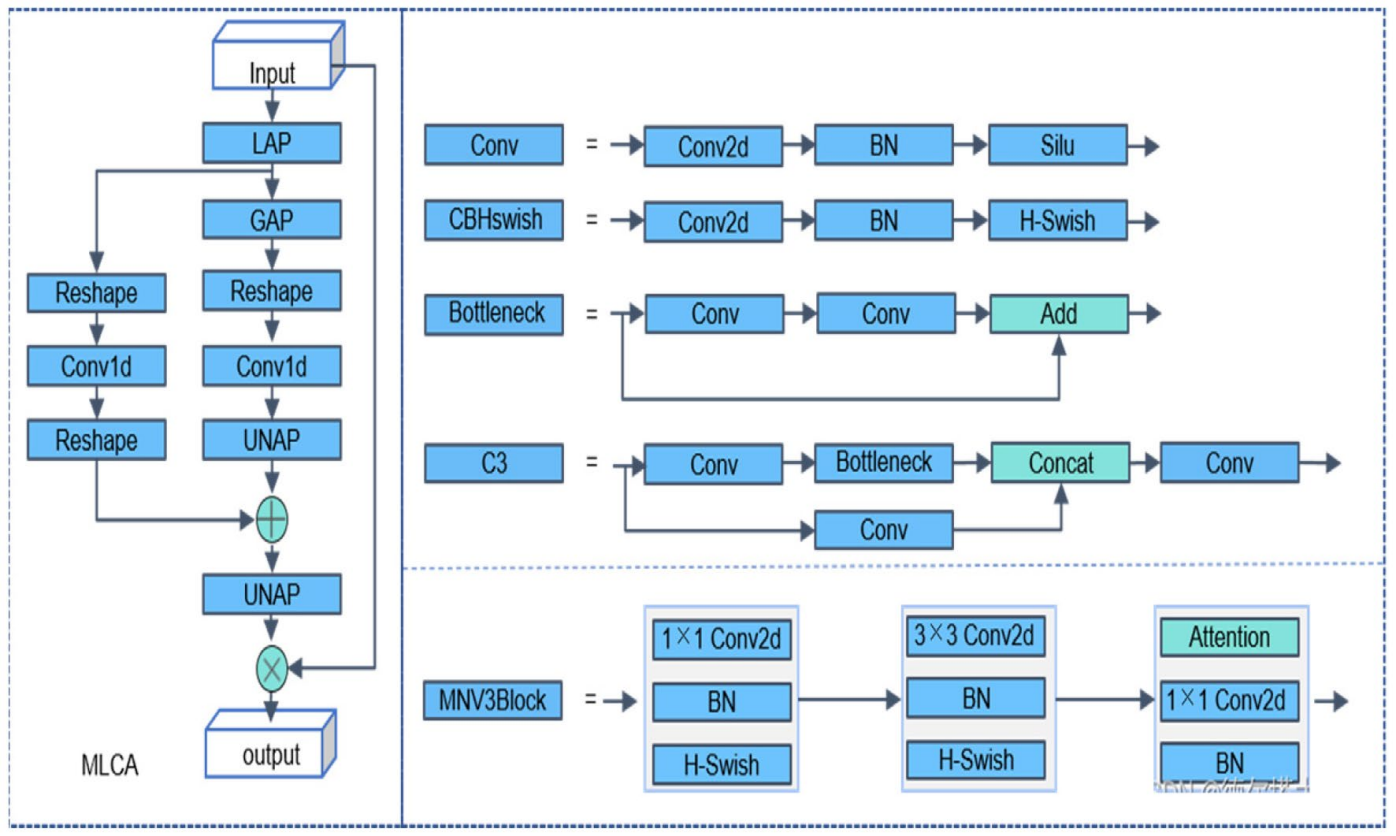
Attention mechanism structure of MLCA mixed local channels.

The C3k2 module primarily concentrates on channel feature fusion, which results in insufficient exploitation of spatial information. Consequently, the accuracy and robustness of feature extraction diminish. Furthermore, the C3k2 module's limited capacity for inter‐channel information exchange hinders the effective utilization of complementary information between channels, impeding the precise identification of potato peeling regions. To address these issues, this paper proposes the MLCA (Mixed Local Channel Attention) module (Wan et al. 2023). By partitioning the feature map into smaller blocks and applying a local attention mechanism, the MLCA module effectively preserves key spatial features and compensates for the C3k2 module's spatial information utilization deficiencies. Simultaneously, the MLCA module enhances the feature map's discriminative ability by assigning greater weight to significant features, thereby improving the identification of broken skin areas. Additionally, the MLCA module's lightweight design optimizes the efficiency and accuracy of feature extraction, achieving this without a substantial increase in computational complexity through the use of one‐dimensional convolution and de‐pooling operations. This approach not only enhances the model's detection performance but also sustains high computational efficiency. The MLCA module also further enhances the performance of skin breaking injury detection through local/global feature fusion. Local features can capture the detailed information of the broken skin area, such as texture changes and color differences, and highlight the features related to the damage through feature selection and enhancement mechanisms, thereby locating the damage location more accurately. Global features provide overall context information to help the model determine the relationship between the damaged area and the surrounding normal area and avoid misjudgment. This fusion method enables the model to accurately identify broken skin damage while maintaining high robustness and adaptability, further optimizing the detection efficiency and making it more advantageous in practical applications. Figure 2 illustrates the structure of the MLCA module.

**FIGURE 3:**
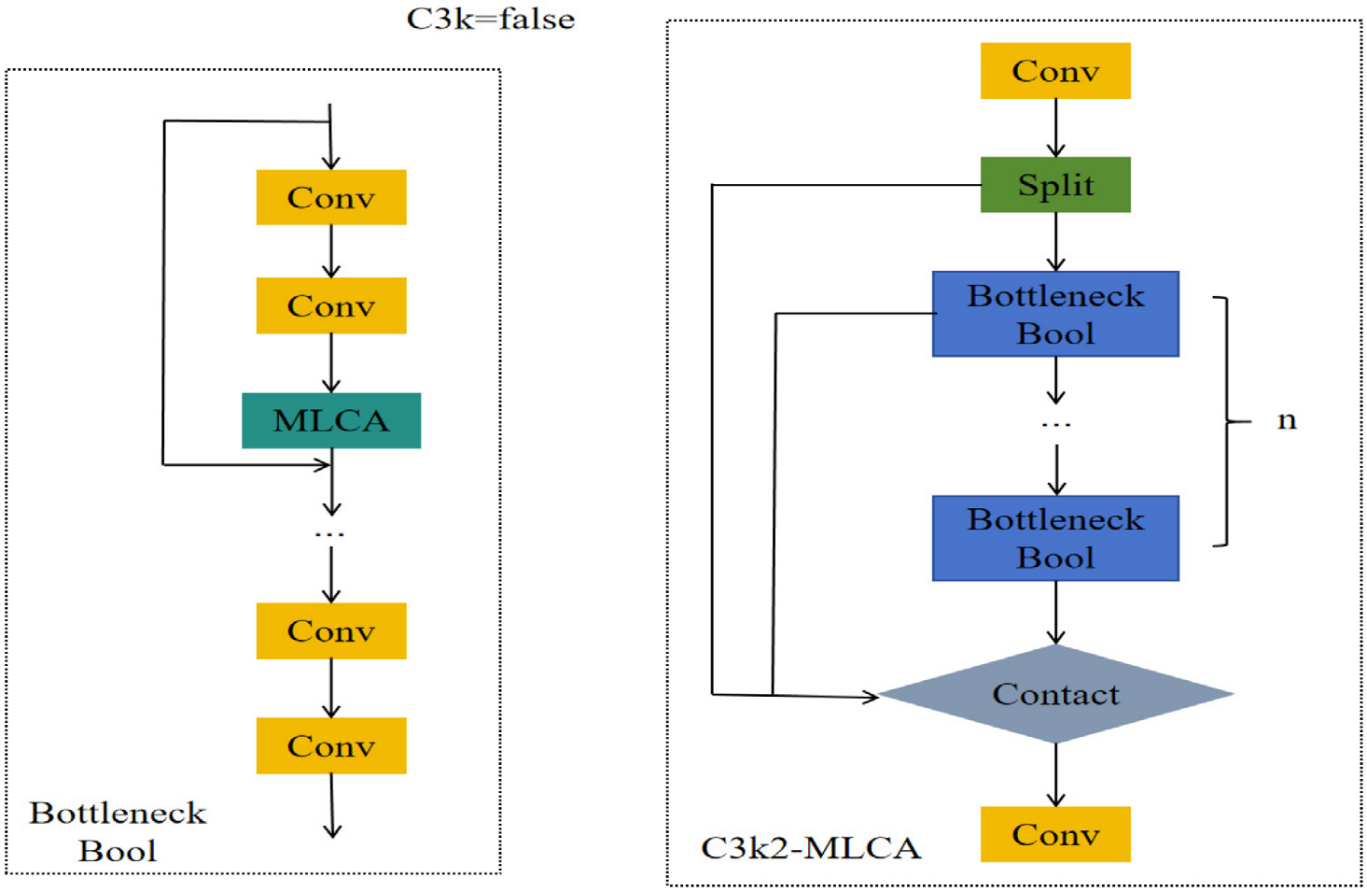
C3k2‐MLCA structure diagram.

The input feature map is initially processed by LAP and GAP to extract local and global features, respectively. These features are subsequently transformed and rearranged through one‐dimensional convolution. Local features are multiplied with the original input features to achieve feature selection and enhancement; global features are added to the local features to integrate global context information. Finally, the processed feature map is restored to its original spatial dimension through de‐pooling. The structure of the constructed C3k2‐MLCA module is illustrated in Figure 3.

#### 2.1.3 ReCalibrationFPN‐P345

**FIGURE 4:**
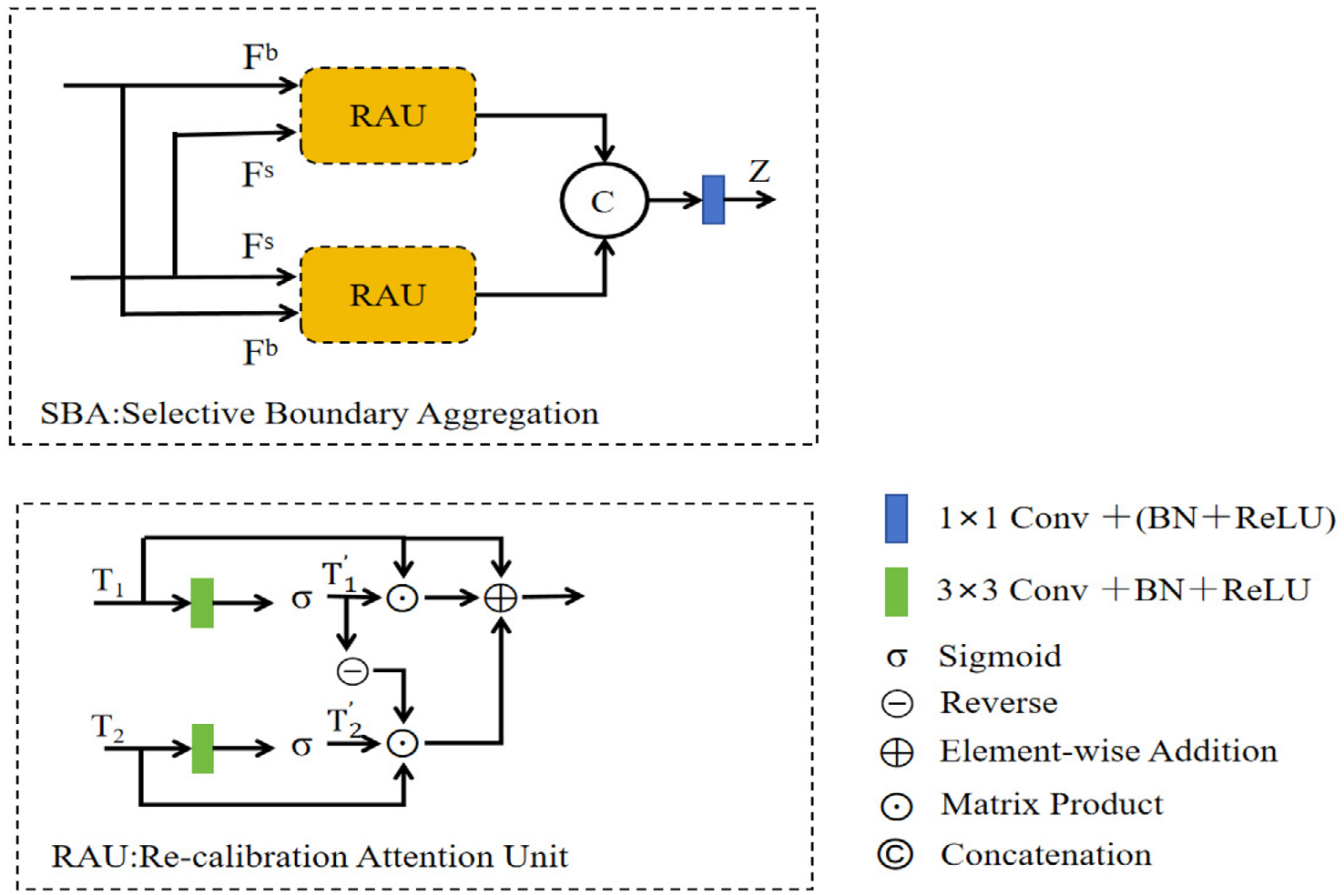
SBA module and RAU structure diagram.

To enhance the interaction between shallow and deep features, the Re‐Calibration FPN is proposed. In the context of detecting broken potatoes, shallow layer features offer rich details and clear boundary information, aiding in the precise localization of the broken skin region. However, they contain limited semantic information. On the other hand, deep features are rich in semantic meaning and can recognize the overall characteristics of the broken skin region. Nonetheless, directly fusing these two types of features can result in redundant and inconsistent information. To address this, the Selective Boundary Aggregation (SBA) module (Tang et al. 2022) is introduced to outline the broken area and recalibrate the location by selectively aggregating boundary and semantic information. Compared to the traditional Feature Pyramid Network (FPN), the SBA module employs a bidirectional fusion mechanism for high‐resolution and low‐resolution features, which improves the efficiency of information transmission. It dynamically adjusts feature weights based on resolution and content through an adaptive attention mechanism, effectively capturing multi‐scale features and enhancing the fusion effect. This better suits the requirements of potato detection tasks and improves detection accuracy. The structure diagram is illustrated in Figure 4.

The RAU block initially performs linear mapping on the input features F^s^ and F^b^ separately, reducing the channel dimension to 32 using 1 × 1 convolution to obtain the dimensionally reduced feature sum. Subsequently, the features are fused through dot multiplication and a reverse operation to refine the rough features and produce more precise prediction graphs. Building upon this, the SBA module employs a bidirectional RAU block design to extract bidirectional features from F^s^ and F^b^ individually, concatenating the outputs along the channel dimensions. Finally, it further fuses these through a 3 × 3 convolution (which includes batch normalization and a ReLU activation layer) to generate output features that encompass both deep semantic information and shallow boundary information.

#### 2.1.4 EIEStem

**FIGURE 5:**
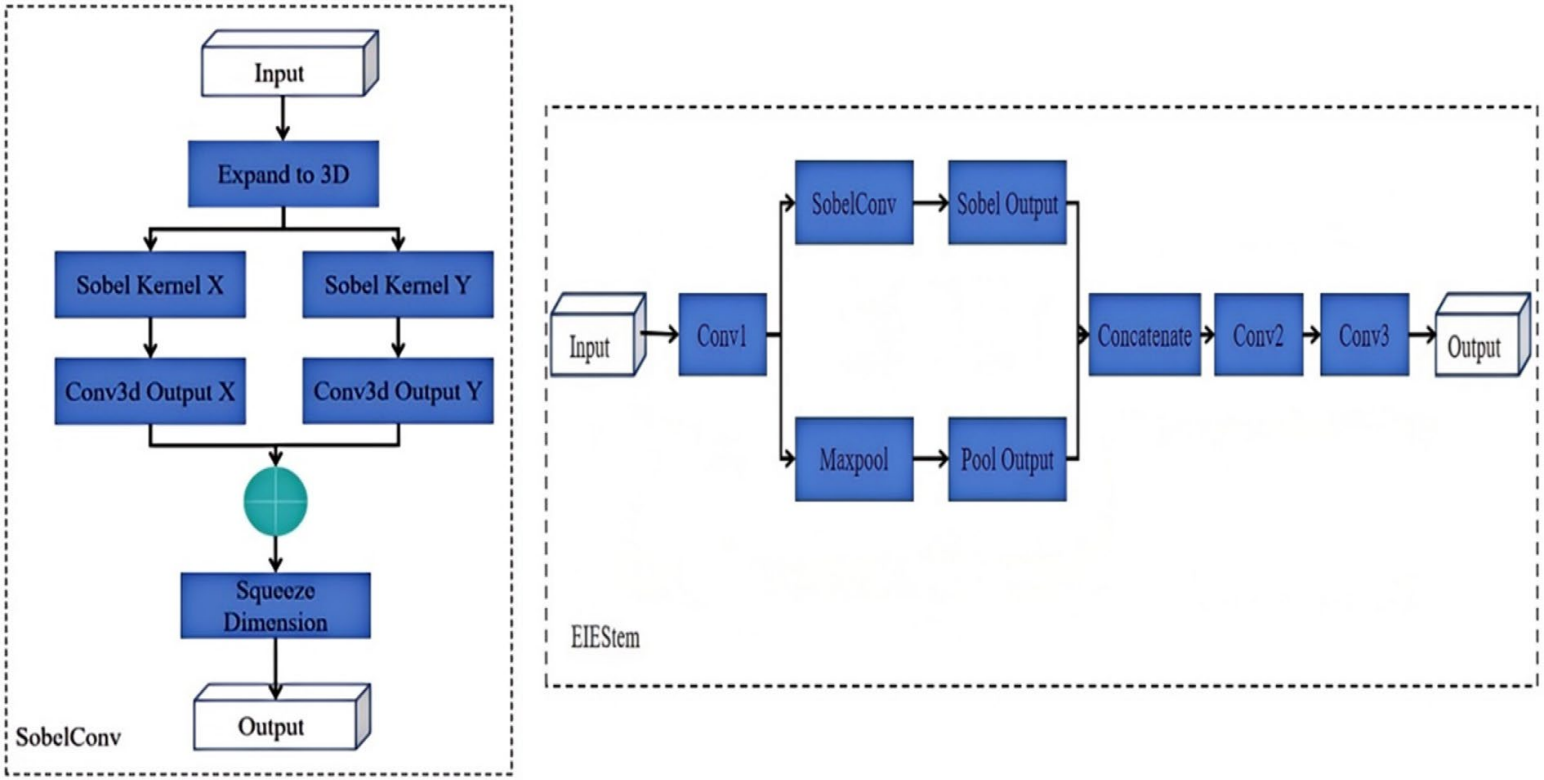
Sobel operator and EIEStem structure diagram.

To enhance the precision of potato peel defect detection, an EIEStem module has been developed and integrated to substitute the initial two convolutional layers of the conventional Backbone network. By incorporating a SobelConv branch for edge information extraction and a standard convolution branch for spatial information, the EIEStem module effectively learns the feature representation of potato images. The SobelConv branch employs the Sobel operator to efficiently extract edge features from the damaged skin area, aiding the model in accurately identifying the location of the defects. Meanwhile, the convolution branch concentrates on extracting the spatial information of the image, thereby enhancing the overall comprehension of the damaged skin area's features. Furthermore, the EIEStem module implements the Sobel operator efficiently through 3D group convolution, optimizing computational efficiency. This architecture not only improves the model's capacity to capture edge features of damaged skin but also leverages multi‐scale features and spatial information, significantly boosting the model's detection accuracy for damaged potatoes while preserving high efficiency. The Sobel operator and EIEStem structure are depicted in Figure 5.

The input features are initially extracted via a convolutional layer, which also reduces the spatial resolution. Subsequently, the module is bifurcated into two branches: one is the Sobel branch, employing SobelConv to extract edge information; the other is the pooling branch, which employs zero‐padding and maximum pooling operations to enhance the robustness of the features. The outputs from both branches are concatenated along the channel dimension, fusing edge information with pooling information. The concatenated features are then further processed by another convolutional layer, which again reduces the spatial resolution. Finally, the number of channels is adjusted using a 1 × 1 convolutional layer to produce the final feature map. With the introduction of the EIEStem module, the model is capable of fully extracting the edge and spatial information of the image at the initial stage, thereby enhancing the expressiveness of the features and aiding in the improvement of broken potato detection accuracy.

#### 2.1.5 AFPN‐P345

**FIGURE 6:**
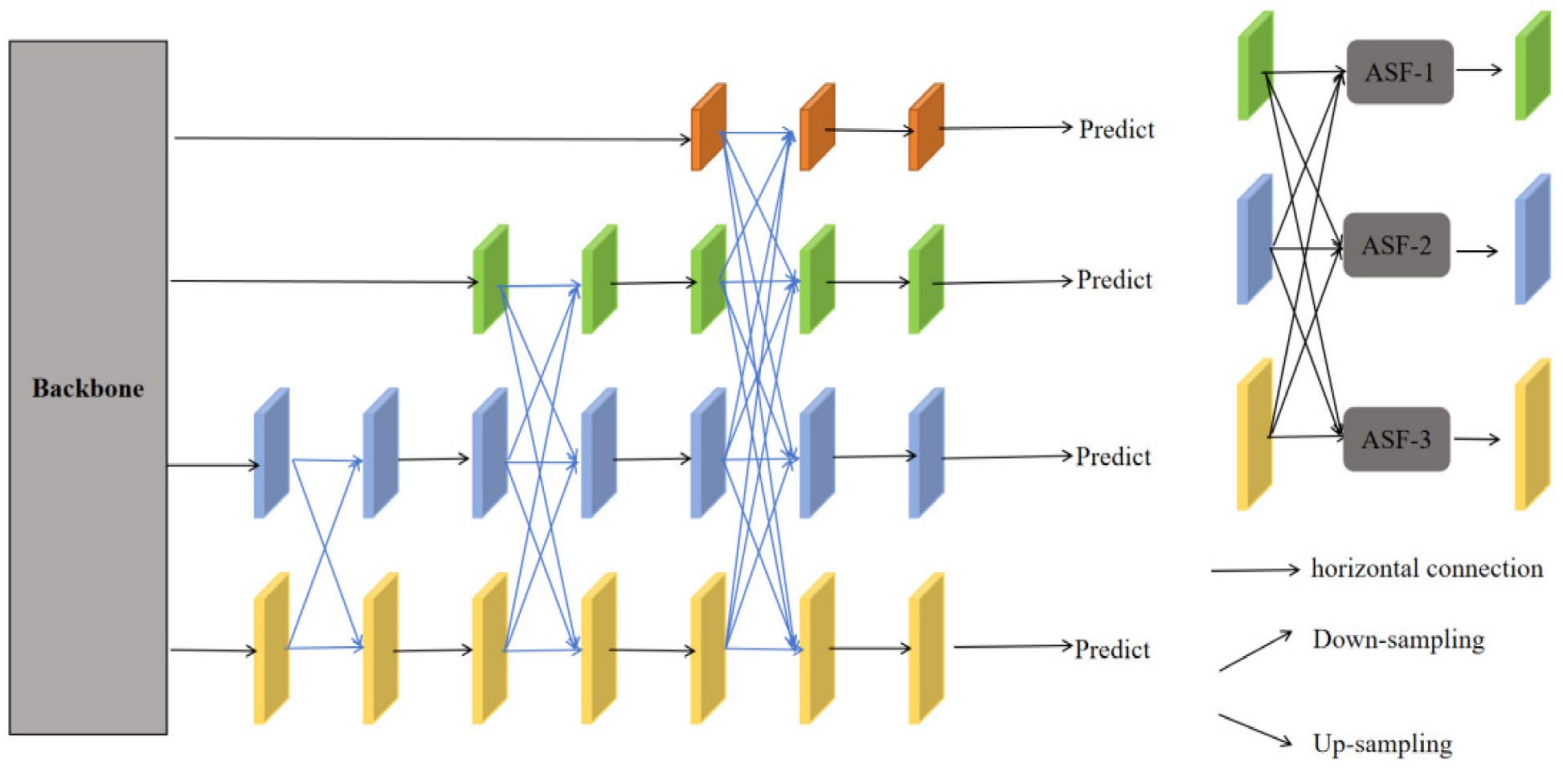
AFPN structure diagram.

In this paper, the Asymptotic Feature Pyramid Network (AFPN) (Yang, Lei, et al. 2023; Yang, Liu, et al. 2023) was utilized to optimize the detection head of YOLOv11, thereby further enhancing the detection performance for potato peel defects. The essence of AFPN lies in the introduction of an asymptotic feature fusion strategy, which progressively merges low‐level and high‐level features into the target detection process. This approach aids in narrowing the semantic gap between features at different levels, enhancing the efficacy of feature fusion, and enabling the detection model to better adapt to varying levels of semantic information. Figure 6 illustrates the AFPN structure.

The input images are extracted by the backbone network to obtain multi‐scale basic features at various resolutions, ranging from high‐level semantic information to low‐level details. Subsequently, the Asymptotic Feature Pyramid Network (AFPN) employs an asymptotic fusion module to progressively integrate features from different levels. This is achieved through top‐down semantic information transfer and bottom‐up detail enhancement. The process is iterative, with each iteration updating the semantic and detailed information of the features. Consequently, the feature pyramid becomes increasingly stable and optimized after several iterations. In the end, the constructed feature pyramid encompasses not only abundant semantic information but also preserves sufficient details.

#### 2.1.6 Loss Function Improvement

YOLOv11n utilizes the CIoU loss function (Zheng et al. 2021) as its foundational loss. This function comprehensively considers the overlap area, center point distance, and aspect ratio difference between target frames, offering certain advantages in processing the geometry of the target, the overlap degree of the boundary frame, and the center distance. However, CIoU does not perform well in processing different target scales, dispersion, or heterogeneity.

To address this issue, the paper presents a novel dynamic non‐monotonic focusing mechanism, Wise‐IoU (WIoU) (Tong et al. 2023), which concentrates on significant regions of the target and dynamically modulates the intensity of attention across various parts based on the target's actual conditions. Its fundamental formulation is as follows:

(1) WIoU=IoU+

WIoU reflects the relationship between targets using a weighted IoU coefficient, which is related to target distance, area, or category. It employs a dynamic focusing mechanism to dynamically weight feature maps based on the spatial location, scale, context, or confidence of targets, effectively enhancing recognition accuracy under complex target distributions.

In model training, low‐quality samples and geometric factors, such as distance and aspect ratio, may affect the model's generalization capabilities. To mitigate this issue, WIoU introduces a distance attention mechanism and constructs WIoU v1 with a two‐tier attention structure.

(2) $$L_{{\text{WIoU}}_{v1}}=R_{\text{WIoU}}L_{\mathrm{IoU}}$$

(3) $$R_{\text{WIoU}}=\exp\left(\frac{{\left(x-x_{\mathrm{gt}}\right)}^{2}+{\left(y-y_{\mathrm{gt}}\right)}^{2}}{{\left(W_{g}^{2}+H_{g}^{2}\right)}^{*}}\right)$$

Among them, the one that significantly enlarges the ordinary quality anchor box and the one that significantly reduces the high quality anchor box, *W*_g_ and *H*_g_ represent the smallest size of the closed box. To prevent the generation of factors that affect convergence, *W*_g_ and *H*_g_ are kept separate in the calculation results (indicated by the superscript).

While WIoU v2 and v3 have shown improved performance, their lengthy training times and high computational complexity do not align with the real‐time and efficiency requirements outlined in this paper. Consequently, this paper focuses solely on the dynamic focusing mechanism of WIoU v1 to enhance the attention and detection capabilities for potato peel breakage areas, thereby reducing instances of missed and false detections.

### 2.2 Dataset Construction and Model Training

#### 2.2.1 Dataset Preparation

In the research on the potato skin breaking detection algorithm, we constructed a sample dataset containing 601 images, covering various types of skin breaking damage. To further enrich the diversity of the dataset, we also artificially produced a portion of samples with specific damage characteristics based on the morphological features of the broken skin. However, due to the complex and diverse forms of skin damage in the actual environment and the influence of multiple factors (such as harvesting methods, transportation conditions, soil moisture, etc.), it is quite difficult to collect a large number of different types of sample images, and it is extremely challenging to completely cover all possible skin damage forms. The number of samples directly affects the training effect of the model. The unprocessed original dataset is difficult to guarantee the accuracy and robustness of the network. Therefore, we implemented data augmentation techniques on the original dataset by using methods such as horizontal flipping, translation, rotation, luminance and contrast adjustment, and scaling. Eventually, a dataset containing 1864 images (including the original images and their augmented images) was generated. Data augmentation not only accelerates the training speed of the model and improves its accuracy, but also helps to reduce overfitting and underfitting problems, thereby enhancing the generalization ability of the model in object detection and image segmentation tasks (Zhu et al. 2021). Ultimately, we divided the dataset into the training set, the test set, and the validation set in an 8:1:1 ratio.

#### 2.2.2 Test Equipment and Environment

The computer environment utilized during the system training phase was based on Python 3.8, PyTorch 1.1.1, a 12th Generation Intel (R) Core (TM) i7‐12700H processor running at 2.30GHz, an Nvidia RTX 3060 graphics card, and 16GB of memory.

In this study, the YOLOv11n network was employed as the foundational model architecture. Throughout the training process, the initial learning rate was established at 0.00001, the final learning rate at 0.0001, and the number of epochs was set to 200. Due to GPU memory constraints, the batch size was configured to 16, the optimizer used was Adam, and the weight decay was set to 0.0005.

#### 2.2.3 Evaluation Indicators

To comprehensively evaluate the performance of the improved algorithm presented in this paper, we utilize widely accepted metrics in object detection, including the mean Average Precision (mAP), precision (P), recall (R), and F1 score. Additionally, we consider Frames Per Second (FPS), model size, and the number of parameters as evaluation criteria. The F1 score is a balanced measure of precision and recall, while mAP assesses the model's detection accuracy. Model size and the number of parameters gauge the model's level of optimization for resource efficiency; FPS indicates the speed of detection, with higher values corresponding to faster processing. The mAP error margins show the fluctuation of the model performance, and the FPS error margins reflect the stability of the system operation. The specific calculations for these indicators are as follows:

(4) $$\mathrm{AP}=\int_{0}^{1}P\;\left(R\right)\;\mathrm{dR}$$

(5) $$\mathrm{mAP}=\frac{\sum_{i=1}^{N}AP_{i}}{N}$$

(6) $$P=\frac{N_{\mathrm{TP}}}{N_{\mathrm{TP}}+N_{\mathrm{FP}}}\times 100\%$$

(7) $$R=\frac{N_{\mathrm{TP}}}{N_{\mathrm{TP}}+N_{\mathrm{FN}}}\times 100\%$$

(8) $$F1=2\frac{\left(\mathrm{PR}\right)}{\left(P+R\right)}$$

(9) $$\mathrm{SD}=\sqrt{\frac{1}{n-1}\sum_{i=1}^{n}{\left(x_{i}-\bar{x}\right)}^{2}}$$

(10) $$\mathrm{CI}=\bar{x}\pm z\times \frac{\mathrm{SD}}{\sqrt{n}}$$

where, *N*_TP_ signifies that the model accurately predicts a positive sample as positive; *N*_FP_ indicates that the model incorrectly predicts a negative sample as positive. *N*_FN_ represents the scenario where the model incorrectly predicts a positive sample as negative; *x*_i_ represents the mAP/FPS value of each experiment, $\bar{x}$ represents the average value of mAP/FPS, *n* represents the number of experiments, and is taken as 5; *z* is the critical value of the standard normal distribution. For the 95% confidence interval, *z* is approximately 1.96.

## 3 Results and Discussion

### 3.1 Comparative Experiment of Loss Function

**FIGURE 7:**
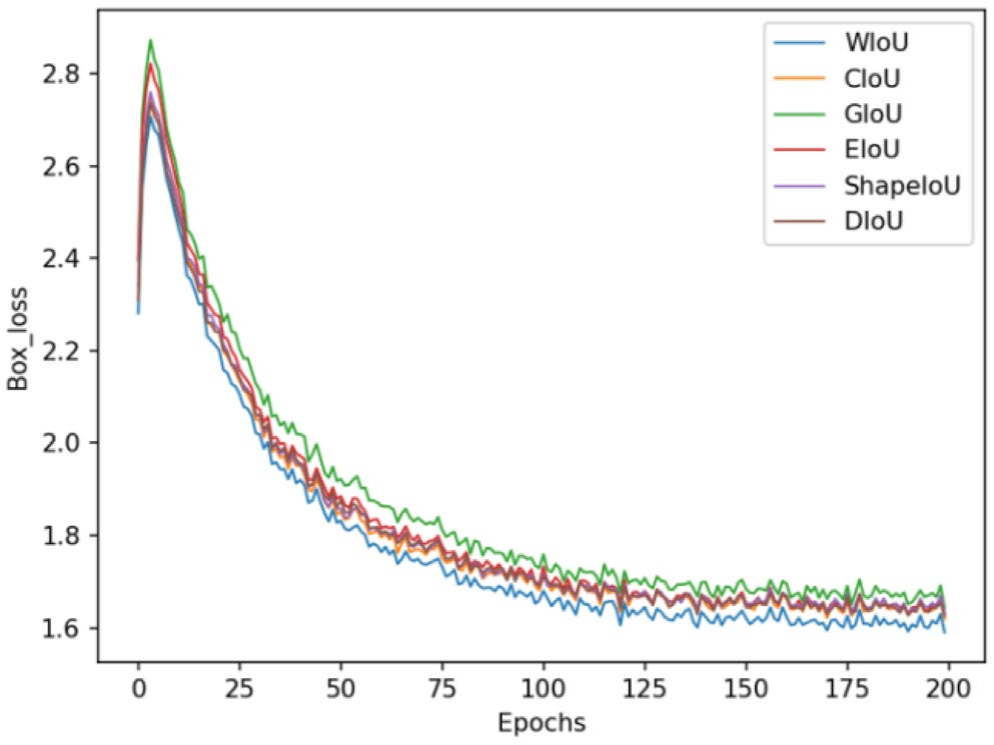
Loss value change curves of different loss functions.

This study employs the basic model YOLOv11n to conduct experiments with six distinct loss functions: CIoU loss, DIoU loss (Zheng et al. 2021), GIoU loss (Rezatofighi et al. 2019), WIoU loss, EIoU loss (Zhang et al. 2022), and ShapeIoU loss (Zhang and Zhang 2023). All six loss functions were evaluated using the potato peel image dataset, with the respective outcomes depicted in Table 1 and Figure 7.

**TABLE 1:** Comparison of different loss functions.

Model	Bounding box regression loss	mAP@0.5 (%)	mAP@0.5:0.95 (%)	Size (MB)	Params (106)
YOLOv11n	CIoU	90.1	44.4	6.3	2.58
	GIoU	87.6	42.3		
	EIoU	90.6	43.6		
	ShapeIoU	89.6	43.1		
	DIoU	89.7	43.6		
	WIoU	92.6	46.4		

According to the quantitative results in Table 1, compared with CIoU loss, GIoU loss experienced a decrease of 2.5% in mAP@0.5 and 2.1% in mAP@0.5:0.95. EIoU loss saw an increase of 0.5% in mAP@0.5, but a decrease of 0.8% in mAP@0.5:0.95; ShapeIoU loss decreased by 0.5% in mAP@0.5 and 1.3% in mAP@0.5:0.95; DIoU loss decreased by 0.4% in mAP@0.5 and 0.8% in mAP@0.5:0.95. Due to its ability to effectively address the boundary box position error by weighting the overlapping area, WIoU achieved the highest average accuracy rate in the loss function comparison experiment presented in this paper. Specifically, on the potato peel breaking image dataset, WIoU loss outperformed CIoU loss by 2.5% in mAP@0.5 and by 2% in mAP@0.5:0.95.

The six models presented in Table 1 are subjected to qualitative analysis in Figure 7. It is evident that the model achieves convergence through training with the data set. Within the potato broken skin image data set, the final loss value for GIoU loss is approximately 1.7, while the final loss values for CIoU loss, ShapeIoU loss, DIoU loss, and EIoU loss are around 1.68. Notably, the final loss value for WIoU loss decreases to 1.62, indicating that WIoU loss outperforms the other losses.

**FIGURE 8:**
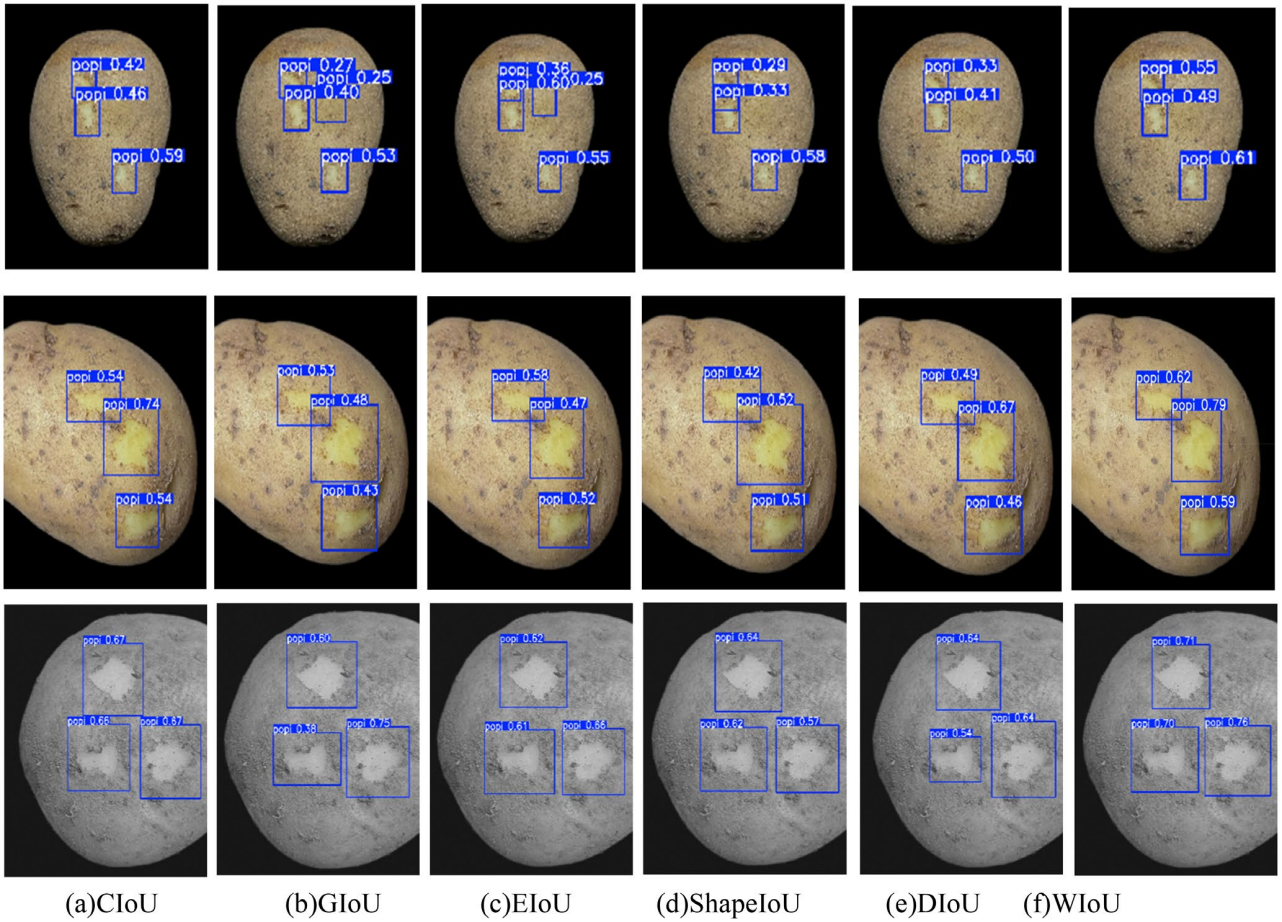
Visualization result diagram.

The visualization results are shown in Figure 8. The average confidence level of the detection box of the WIoU loss function is generally higher than that of other loss functions. This is because the WIoU loss can be dynamically adjusted for the overlapping area by introducing a weighting mechanism. This mechanism makes the model more flexible when dealing with the size and shape changes of bounding boxes and is particularly suitable for the task of potato peeling detection. In this task, the shape and size of the broken skin area vary significantly depending on the degree of damage. WIoU loss can dynamically monitor these changes, thereby more accurately locating and identifying the broken skin area.

In summary, the WIoU loss demonstrates exceptional positioning accuracy. Consequently, in this paper, the WIoU loss is chosen as the fundamental loss function for subsequent experiments.

### 3.2 Comparison Experiment With Mainstream Algorithms

**FIGURE 9:**
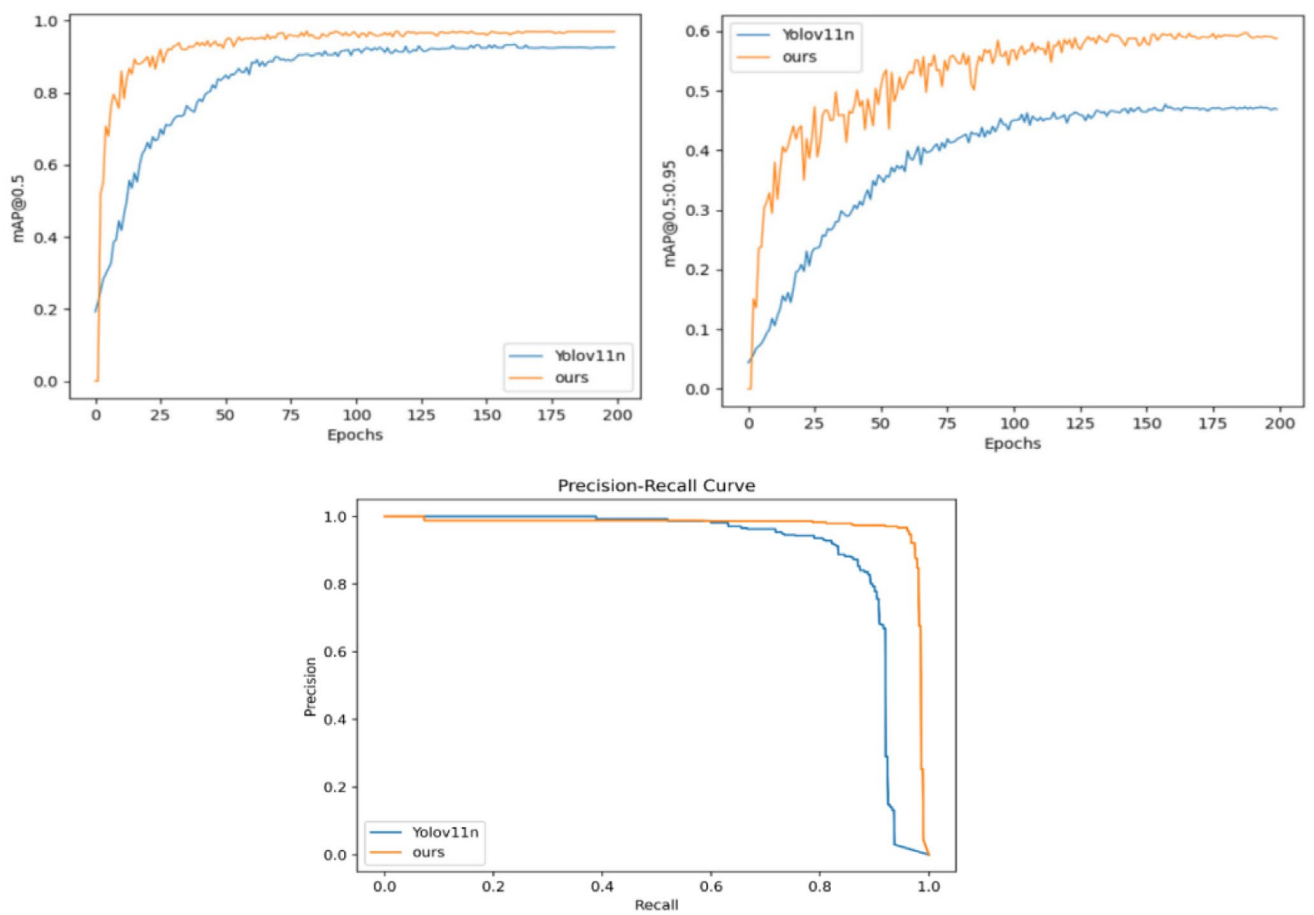
Comparison of performance indicators before and after improvement.

To demonstrate the superior performance of the improved algorithm model presented in this paper, we conducted a qualitative comparison with current target detection algorithms: YOLOv10s (Wang, Chen, et al. 2024; Wang, Yeh, et al. 2024), YOLOv10n, YOLOv9t (Wang, Chen, et al. 2024; Wang, Yeh, et al. 2024), YOLOv8n, YOLOv8‐P2, Yolov8N‐Ghost, and YOLOv6n (Li et al. 2022), YOLOv5n, Yolov5N‐P6, and YOLOv3‐tiny (Adarsh et al. 2020). To ensure consistency, all these networks were trained using the same dataset, and after training, the same test set was employed for evaluation. The detection results were assessed using a unified standard. The quantitative results are presented in Table 2, while the qualitative results are depicted in Figure 9.

**TABLE 2:** Comparative experimental results.

Experiment	*p* (%)	r (%)	mAP@0.5 (%)	mAP@0.5:0.95 (%)	mAP error margins	F1	FPS	FPS error margins	Size (MB)	Parameters (10^6^)
YOLOv11n	89.1	84.2	90.1	44.4	±0.105	0.866	303.03	±0.318	6.3	2.58
YOLOv10s	77.9	81.1	85.8	45.7	±0.089	0.794	217.39	±0.217	21.4	7.22
YOLOv10n	69.1	71	76.4	32.9	±0.076	0.70	181.82	±0.182	6.5	2.26
YOLOv9t	89.9	85.7	90.6	44.8	±0.112	0.877	256.41	±0.356	6.4	1.73
YOLOv8n	91.7	83.6	90.1	43	±0.098	0.875	270.27	±0.370	6.8	2.68
YOLOv8‐P2	93	88.1	92	41.2	±0.102	0.905	120.48	±0.120	10.9	2.77
YOLOv8n‐ghost	85.1	76.1	78.5	29.5	±0.067	0.803	138.90	±0.139	3.8	1.39
YOLOv6n	88.4	88.1	91.1	45.6	±0.083	0.882	138.90	±0.148	11.5	4.15
YOLOv5n	91.6	86.4	88.4	44.5	±0.091	0.889	147.06	±0.157	5.8	2.18
YOLOv5n‐P6	91.8	86.5	90.1	42.6	±0.087	0.891	114.94	±0.134	5.9	3.68
YOLOv3‐tiny	92.7	86	87.6	42.1	±0.074	0.892	147.06	±0.167	14.3	9.52
(ours)	96.5	96.0	97.0	59.4	±0.058	0.962	125	±0.105	16.1	5.87

^a^ The error ranges in the tables are represented as 95% confidence intervals, and the calculation is based on the standard deviation of multiple experiments.

As shown in Table 2, the proposed algorithm has significant advantages in detection accuracy. Its mAP@0.5 reaches 97%, the highest among all algorithms. Meanwhile, its mAP@0.5:0.95 is 59.4%, second only to YOLOv10s's 45.7%. The precision (P) and recall (R) are 96.5% and 96%, respectively, with an F1 score of 0.962. These metrics indicate that the algorithm's overall performance is superior to other algorithms while maintaining a good balance between precision and recall. In terms of detection speed, the algorithm's FPS is 125. Although not the fastest, this speed is commendable given its high accuracy. Compared to YOLOv11n and other high frame rate algorithms, the proposed algorithm achieves real‐time performance of 125 FPS without compromising accuracy, meeting the requirements of most real‐time detection tasks and demonstrating a favorable balance between speed and accuracy. Regarding lightweight design, the algorithm performs well. Its model size is 16.1 MB, not the smallest, but its parameter count is only 5.87 × 10^6^, which is relatively low for a high‐precision algorithm.

As illustrated in Figure 9, the proposed algorithm demonstrates a significant enhancement in performance compared to the baseline YOLOv11n algorithm for potato broken skin detection tasks. Specifically, the proposed algorithm achieves a mean Average Precision (mAP) at 0.5 of 97%, whereas YOLOv11n reaches only 90.1%, marking an improvement of 6.9%. Additionally, the mAP at 0.5:0.95 has increased by 15%, from 44.4% to 59.4%. Simultaneously, the accuracy rate (P) and recall rate (R) have attained 96.5% and 96.0%, respectively, which represent an increase of 7.4% and 11.8% over YOLOv11n's 89.1% and 84.2%. These results validate the effectiveness of the optimization strategy presented in this paper. In practical agricultural detection applications, this optimization significantly boosts detection accuracy and efficiency, reduces false and missed detections, and offers more reliable technical support for potato broken skin detection.

In summary, the proposed algorithm achieves an optimal balance between high precision, real‐time performance, and lightweight design. It excels in detection accuracy with the highest mAP@0.5 and F1 scores, and delivers real‐time performance at 125 FPS. Additionally, it performs well in lightweight design with a reasonable model size and parameter count, considering the balance between performance and resource consumption. Overall, it demonstrates significant comprehensive advantages.

### 3.3 Ablation Experiment

**FIGURE 10:**
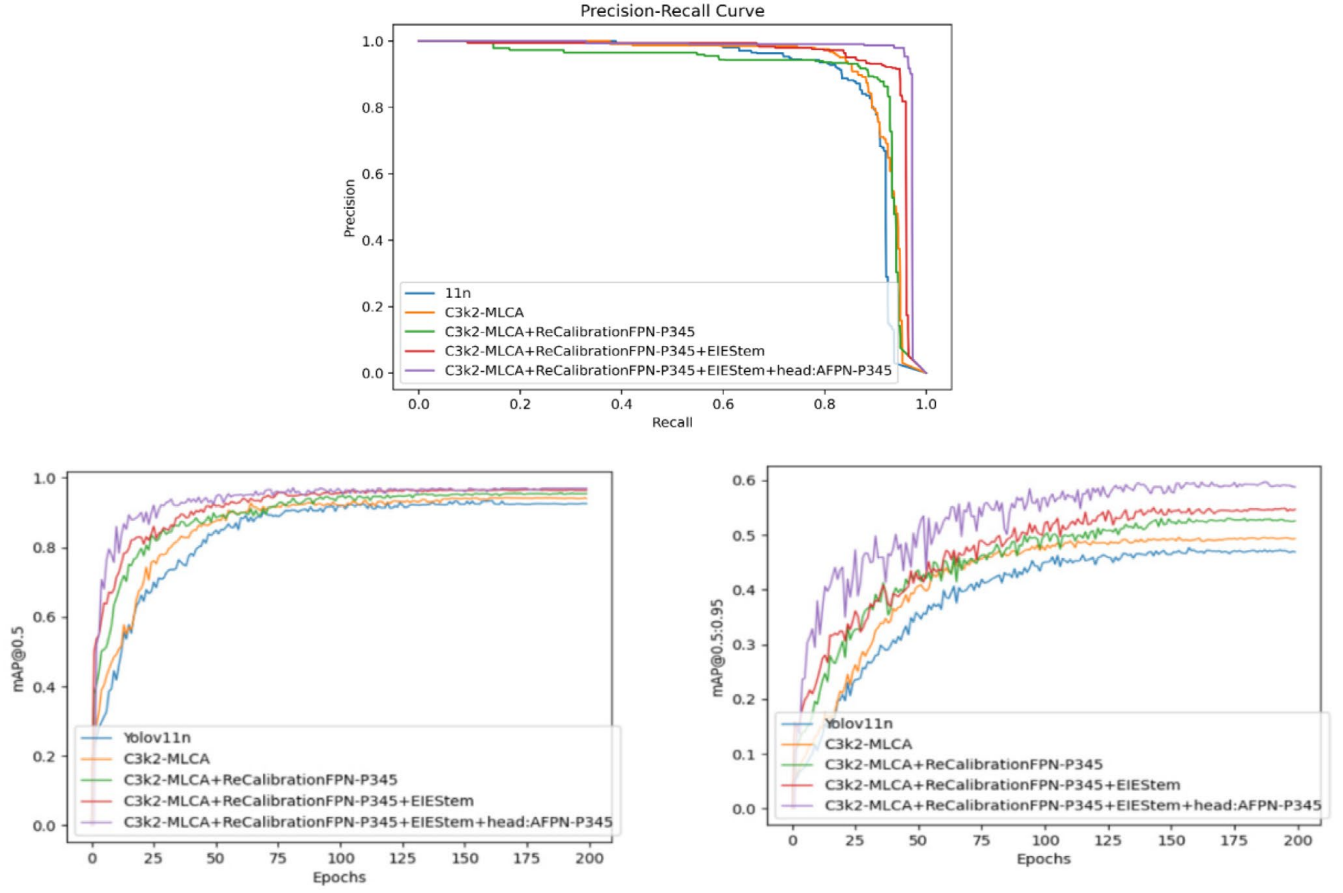
Ablation results of different modules.

To explore the effectiveness of each module and its specific impact on the overall model performance, we conducted a series of ablation experiments on the potato peel dataset. The experiments were divided into five groups: the first group utilized only YOLOv11n; the second group enhanced the C3k2 module of the backbone, building upon the first group; the third group further improved the neck part, based on the enhancements of the second group; the fourth group introduced the EIEStem module into the backbone, following the third group's improvements. The fifth group added a detection head, specifically AFPN‐P345, on top of the fourth group's setup. All experiments in this paper employed uniform training parameters. This paper presents both qualitative analysis, as depicted in Figure 10, and quantitative analysis, as outlined in Table 3.

**TABLE 3:** Results of ablation experiment.

Experiment	*p* (%)	*R* (%)	mAP@0.5 (%)	mAP@0.5:0.95 (%)	mAP error margins	F1	FPS	FPS error margins	Size (MB)	Parameters (10^6^)
v11n	89.1	84.2	90.1	44.4	±0.105	0.866	303.03	±0.318	6.3	2.58
v11n+C3k2‐MLCA	93.9	89.5	94.2	49.5	±0.098	0.916	312.5	±0.295	6.3	2.58
v11n+C3k2‐MLCA+ReCalibrationFPN‐P345	93.4	92.2	95.4	53.1	±0.102	0.928	161.29	±0.234	13.5	3.51
v11n+C3k2‐MLCA+ReCalibrationFPN‐P345+EIEStem	94.6	92.4	96.4	54.9	±0.087	0.935	135.14	±0.198	13.7	3.51
v11n+C3k2—MLCA + ReCalibrationFPN—P345+EIEStem + head: AFPN—P345	96.5	96.0	97.0	59.4	±0.058	0.962	125	±0.105	16.1	5.87

^a^ The error ranges in the tables are represented as 95% confidence intervals, and the calculation is based on the standard deviation of multiple experiments.

Figure 10 illustrates the curve of the mean Average Precision (mAP) value change with the number of training Epochs for each model, along with Precision‐Recall (P‐R) curves depicting accuracy and recall rates. The figure reveals that as each optimization module is incrementally added, the mAP value, accuracy rate, and recall rate of the model exhibit an upward trend. In particular, the performance of the initial model begins to enhance after the first module is incorporated, and with each subsequent module added, the mAP value, accuracy, and recall rate further improve. Ultimately, upon the integration of all optimization modules, the model's performance attains its optimal state, with the mAP value, accuracy rate, and recall rate peaking. This demonstrates that the synergistic effect of the modules is crucial for enhancing model performance and confirms the effectiveness and rationality of the optimization strategy.

As indicated by Table 3, the inclusion of each module notably enhances the model's performance. The detailed analysis is as follows:

1. The integration of the C3k2‐MLCA module into the v11n model significantly enhanced performance. Detection accuracy metrics improved: mAP@0.5 rose from 90.1% to 94.2%, mAP@0.5:0.95 from 44.4% to 49.5%, precision (P) from 89.1% to 93.9%, recall (R) from 84.2% to 89.5%, and F1 score from 0.866 to 0.916. The module improved potato peel defect detection through multi‐scale feature fusion and attention mechanisms, enhancing precision and localization. Detection speed increased, with FPS rising from 303.03 to 312.5. Notably, the model size (6.3 MB) and parameter count (2.58 × 10^6^) remained unchanged, demonstrating that the C3k2‐MLCA module boosts performance without adding complexity, offering practicality and efficiency;
2. Introduction of the ReCalibrationFPN‐P345 Module: The ReCalibrationFPN‐P345 module enhances the model's feature extraction capabilities, resulting in improved detection metrics: mAP@0.5 reaches 95.4%, mAP@0.5:0.95 increases to 53.1%, precision (P) to 93.4%, recall (R) to 92.2%, and the F1 score to 0.928. This indicates that the recalibrated feature pyramid network can effectively delineate potato peel lesions through bidirectional fusion and adaptive attention, thereby improving multi‐scale feature fusion and better meeting the detection requirements. However, the integration of this module also leads to some trade‐offs: the detection speed (FPS) decreases from 312.5 to 161.29, the model size grows from 6.3 to 13.5 MB, and the parameter count increases from 2.58 to 3.51 million. Specifically, since the SBA module improves the detection accuracy by bidirectionally fusing high and low resolution features, it increases the computational load of convolution operations and feature stitching. Although the bidirectional feature extraction and adaptive attention mechanism of the RAU unit can optimize the feature weights, they also bring additional computational burdens. Meanwhile, the increase in the number of model parameters leads to an increase in memory usage, limiting the parallel computing capability of the hardware. Overall, while the module significantly improves accuracy, it does so at the expense of reduced detection speed and increased model complexity;
3. After integrating the EIEStem module, the model's input module has been optimized. The mean Average Precision (mAP) at 0.5 has risen to 96.4%, while the mAP at 0.5:0.95 has increased to 54.9%. Accuracy (P) has improved to 94.6%, recall (R) has climbed to 92.4%, and the F1 score has increased to 0.935. These enhancements demonstrate that the EIEStem module effectively extracts features from input images of potato peel damage, thereby enhancing the model's detection accuracy. Despite a reduction in detection speed, with FPS dropping from 161.29 to 135.14, the overall performance indicates that the module significantly contributes to the model's accuracy, outweighing the decrease in speed.
4. After integrating the AFPN‐P345 module, the model's high‐precision detection capabilities improved significantly. The mAP@0.5 reached 97%, mAP@0.5:0.95 increased to 59.4%, precision (P) was 96.5%, recall (R) was 96.0%, and the F1 score was 0.962. The adaptive feature pyramid network effectively detected potato peel damage across various scales. However, the detection speed dropped to 125 FPS, the model size grew to 16.1 MB, and the parameter count rose to 5.87 × 10^6^. Despite increased complexity and slower inference time, the model achieved a good balance between accuracy, speed, and compactness, making it suitable for high‐precision detection tasks.

Overall, the gradual addition of each module significantly enhances the model's detection accuracy. Despite slight increases in complexity and inference time, the substantial improvement in mAP is highly valuable.

### 3.4 Visual Analysis of Experimental Results

**FIGURE 11:**
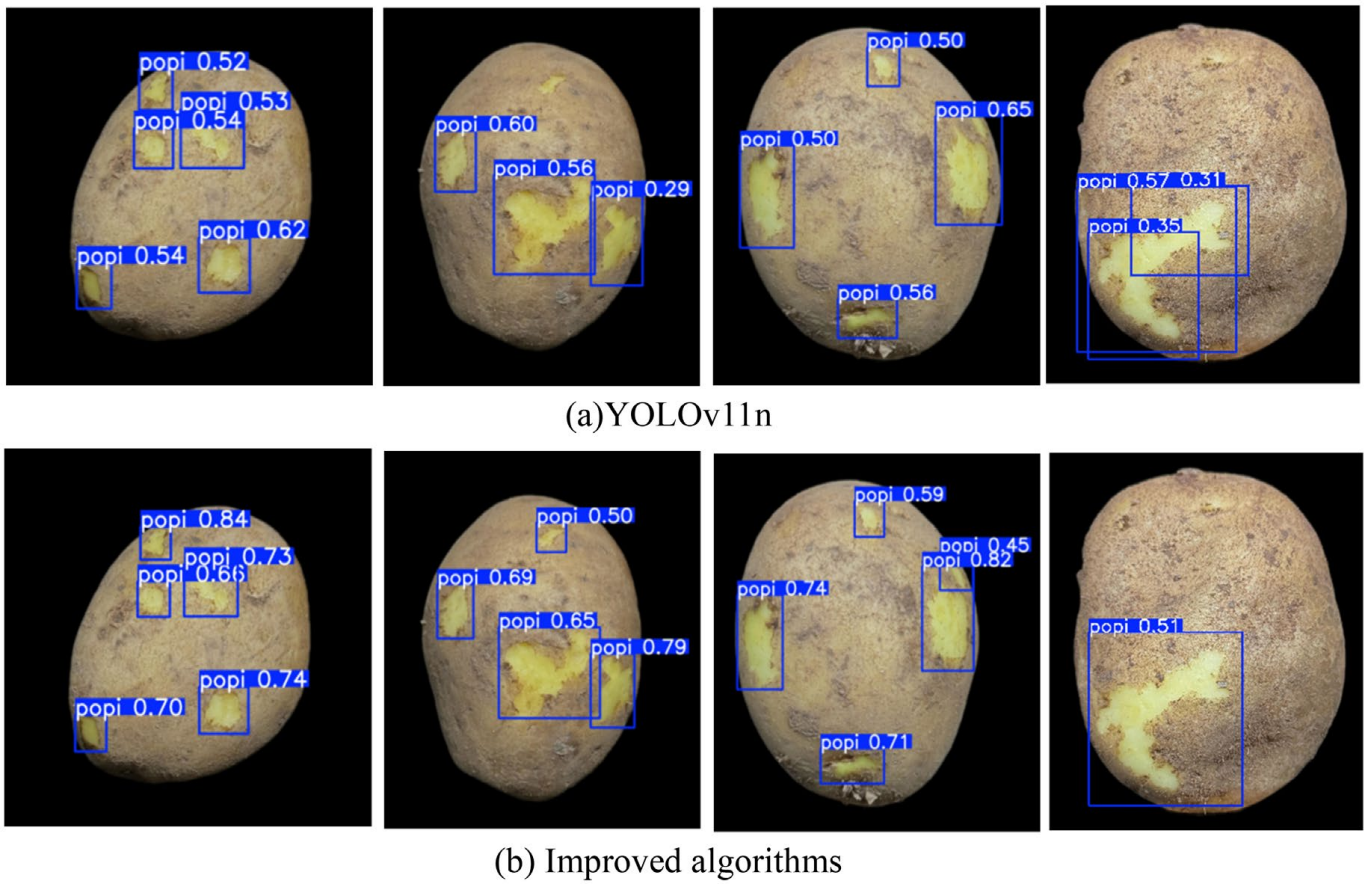
Visual comparison between YOLOv11n algorithm and improved algorithm.

**FIGURE 12:**
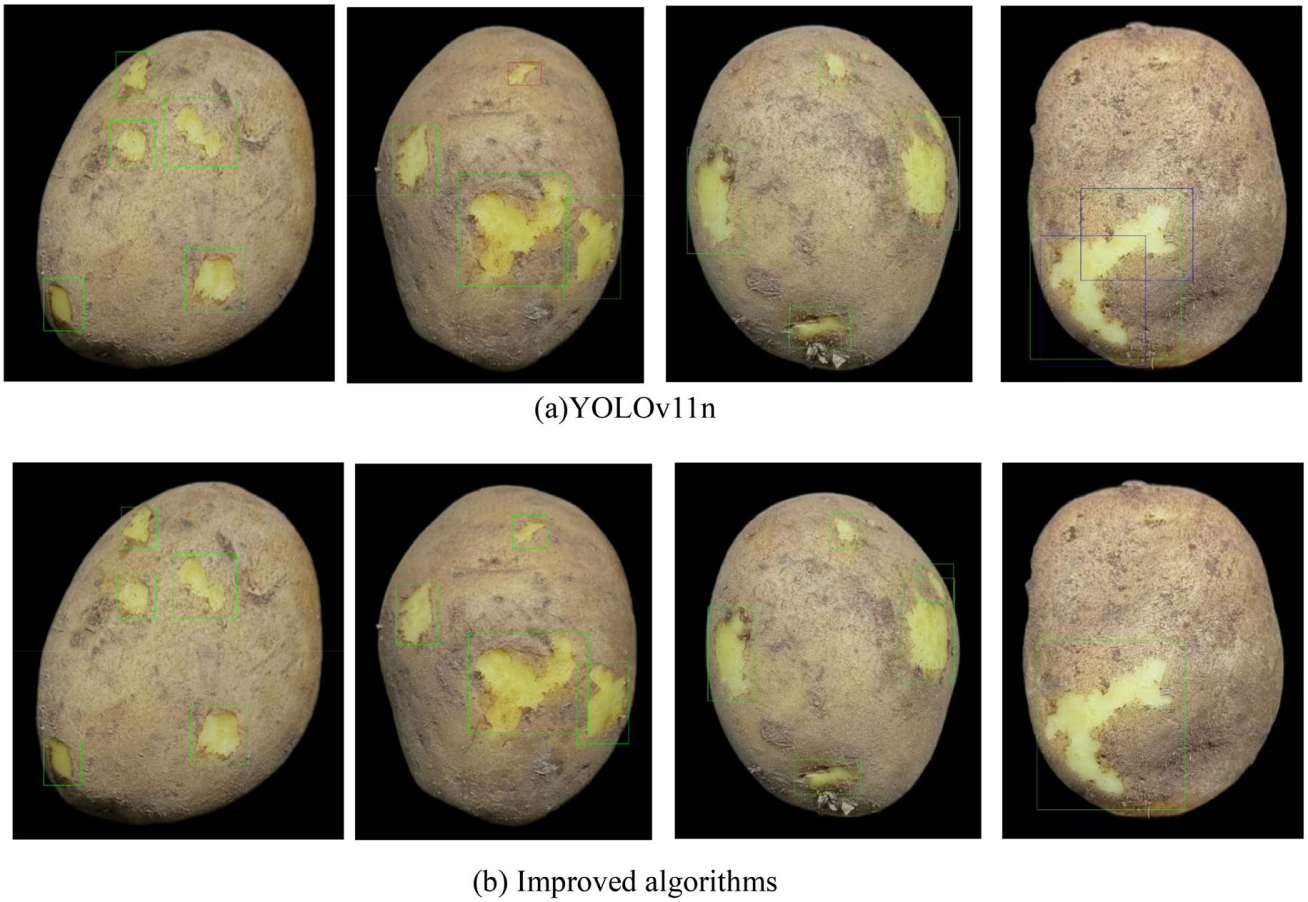
Visualize and statistically analyze TP, FP, and FN in object detection.

To more intuitively evaluate the target detection effect of the improved algorithm, several representative pictures were randomly selected from the potato peel dataset for visual comparison, as shown in Figures 11 and 12.

It can be known from Figures 11 and 12 that there are some problems with the YOLOv11n algorithm when detecting the potato skin breaking target. For targets located at the edge or with smaller dimensions, the YOLOv11n is prone to missed detections (as shown in the red box in Figure 12a), as well as false detections and repeated box selections (as shown in the blue box in Figure 12a). This limitation primarily stems from YOLOv11n's constrained detection capabilities when processing small targets or complex scenes, particularly in feature extraction and confidence evaluation. In contrast, the enhanced algorithm presented in this paper optimizes these areas and effectively prevents the occurrence of missed detections. Furthermore, the average confidence level of the detection boxes from the proposed algorithm is generally higher than that of the YOLOv11n algorithm. This indicates that the proposed algorithm not only identifies targets more accurately but also outputs detection results with greater confidence, thereby demonstrating higher reliability and robustness in practical applications.

**FIGURE 13:**
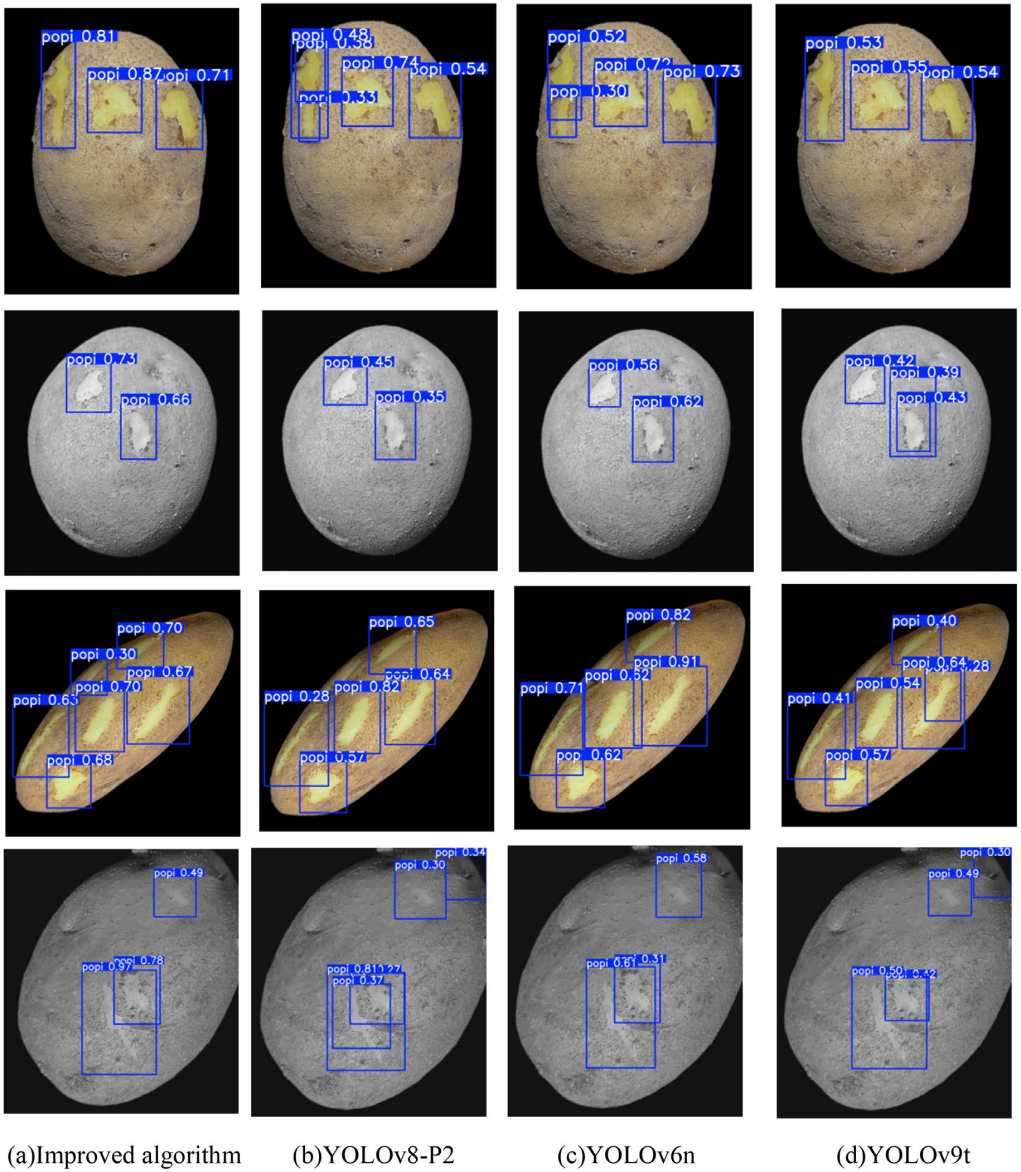
mAP@0.5 Comparison of the top four detection effects.

**FIGURE 14:**
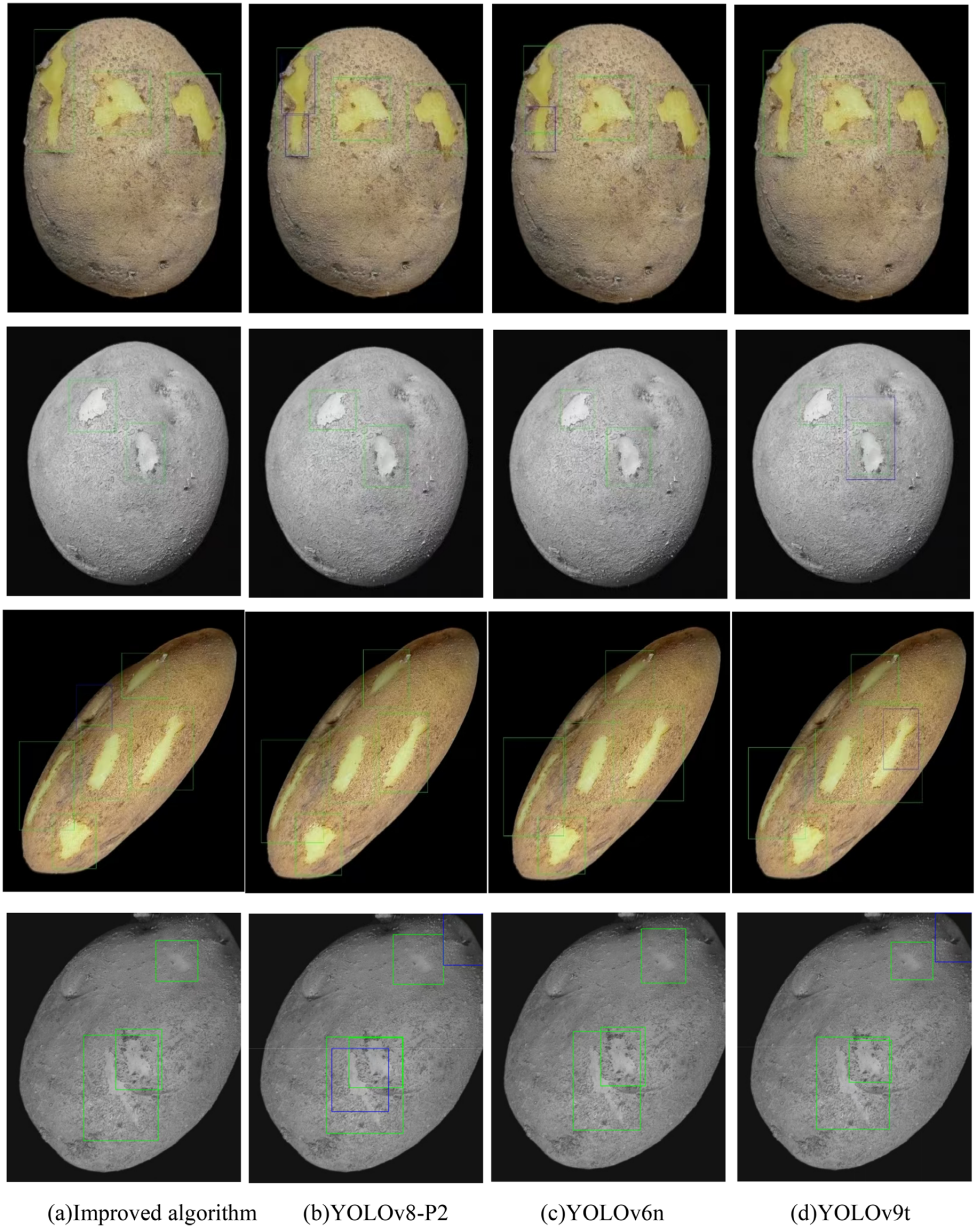
Visualize and statistically analyze TP, FP, and FN in object detection.

Additionally, this paper conducts a visual comparative analysis of the top four models (this paper's algorithm, YOLOv8‐P2, YOLOv6n, and YOLOv9t) ranked by mAP@0.5, as depicted in Figures 13 and 14. To illustrate the performance of each model under various image processing conditions, the original image, grayscale image, stretched image, and cropped image of potato peels were chosen as comparative samples. Through these varied image samples, the disparities among the models in detection accuracy, target location accuracy, and robustness are thoroughly examined, thereby fully showcasing the superiority of the proposed algorithm.

As illustrated in Figures 13 and 14, the improved algorithm presented in this paper exhibits significant advantages in detection effectiveness. Unlike the other three algorithms, which suffer from issues such as repeated box selection, missed detections (as shown by the blue box in Figure 14) and low average confidence of detection boxes, the algorithm proposed in this paper does not experience these problems. The outstanding performance of the algorithm can be primarily attributed to the following factors:

1. The MLCA attention mechanism has been introduced into the C3k2 module to enhance the network's capability to capture key features by integrating channel and spatial attention at both local and global levels. Visualization results indicate that the improved model can more accurately focus on the broken skin area and effectively enhance the confidence of the detection frame.
2. A proposed Re‐Calibration FPN (Feature Pyramid Network) is designed to accurately delineate the contour of potato peels through biometric fusion and an adaptive attention mechanism. The visualization results demonstrate that the accuracy and adaptability of the detection framework are significantly improved when processing skins of varying sizes. For small areas of broken skin, the detection framework can adapt to its actual shape, thereby reducing the occurrence of false and missed detections. For larger areas of broken skin, the framework can be precisely selected to avoid the issue of detection frames being too large or too small.
3. By integrating the SobelConv branch for edge information extraction with the convolution branch for spatial information extraction, the EIEStem module can more effectively learn the feature representation of potato broken skin images. Visualization results indicate that the enhanced model can precisely identify the contours of damaged skin at the edges, and the detection frames align closely with the actual shapes, marking enhancing the accuracy of edge damage detection.
4. The Asymptotic Feature Pyramid Network (AFPN) was utilized to optimize the detection head of YOLOv11, which assisted in narrowing the semantic gap between features at various levels and enhanced the feature fusion effect. Visualization results indicated that the detection model could precisely identify the broken parts of the skin after processing images with stretching and grayscale transformation, without any occurrence of repeated box selection.
5. The WLoU loss function was introduced to address the issue that the traditional CIoU loss function overlooks the shape and scale of the target frame, making border regression more accurate. Visual results indicate that the improved detection frame aligns more closely with the actual broken skin location, and the occurrence of false and missed detections is significantly reduced.

In summary, the enhanced target detection algorithm presented in this paper demonstrates robust performance in recognizing broken skin on potatoes. Experimental results indicate that the algorithm accurately identifies damaged areas on the potato surface, with no instances of missed detection across numerous tests, exhibiting excellent detection stability and reliability. By specifically optimizing the foundational YOLOv11n algorithm, detection accuracy has been notably enhanced. Furthermore, when compared to other existing target detection algorithms, particularly under various image processing conditions, the algorithm sustains a superior level of accuracy.

## 4 Conclusions

To enhance the detection accuracy of potato broken skin regions, this paper introduces a potato broken skin detection algorithm based on an improved YOLOv11n. The primary enhancements are as follows: First, the MLCA attention mechanism is integrated into the C3k2 module within the network's Backbone. By combining channel and spatial attention at both local and global levels, the network's capacity to capture potato peeling features is bolstered, thereby effectively enhancing the model's accuracy. Second, a Re‐Calibration FPN is proposed to refine the Neck component of the YOLOv11 network. This network precisely delineates the potato peel breaking region through bidirectional fusion and an adaptive attention mechanism, enhancing the efficacy of multi‐scale feature fusion to better meet the requirements of potato detection tasks. Furthermore, the initial two convolutional layers of the Backbone are substituted with the EIEStem module, which merges the SobelConv branch for edge information extraction with the convolutional branch for spatial information extraction, thus more effectively learning the feature representation of potato peel images. Simultaneously, the Asymptotic Feature Pyramid Network (AFPN) is employed to optimize the detection head of YOLOv11. This refinement aids in reducing the semantic gap between features at various levels, improving the feature fusion effect, and enabling the detection model to better adapt to semantic information across different levels, thereby further enhancing the detection performance of potato broken skin. Lastly, the WloU loss function is introduced to address the issue with the traditional CIoU loss function, which overlooks the shape and scale of the target frame, resulting in more accurate boundary regression and a reduction in the misidentification and false detection of broken skin.

The results of comparison and ablation experiments demonstrate that the improved algorithm outperforms the benchmark model YOLOv11n by 6.9%, 15%, 7.4%, and 11.8% in terms of mAP@0.5, mAP@0.5:0.95, precision rate (P), and recall rate (R), respectively, on the potato peel breaking dataset. The enhanced algorithm's superiority in detecting broken potato peels is thoroughly substantiated. While there remains potential for optimization in individual metrics such as inference speed or model size, its overall performance surpasses that of existing mainstream algorithms. By enhancing the accuracy and efficiency of detection, the improved algorithm effectively diminishes misjudgments and missed detections in broken potato peel identification, offering a more precise solution for this task and further enhancing the reliability of potato broken skin detection.

Future research will focus on further optimizing the computational efficiency of the model through techniques such as model pruning and knowledge distillation to meet the demands for high efficiency and low latency in practical applications. In addition, the adaptability of this module in the real‐time deployment of target tracking and segmentation tasks will also be verified to expand its application scope.

## Author Contributions

**Qiying Li:** conceptualization (equal), data curation (equal), formal analysis (equal), validation (equal), visualization (equal), writing – original draft (equal). **Ke Chen:** investigation (equal), software (equal). **Qian Wang:** investigation (equal), methodology (equal). **Fuxiang Wang:** conceptualization (equal), resources (equal), supervision (equal), writing – review and editing (equal). **Weigang Deng:** conceptualization (equal), funding acquisition (equal), project administration (equal), resources (equal), supervision (equal), writing – review and editing (equal).

## Conflicts of Interest

The authors declare no conflicts of interest.

## Data Availability

The data that support the findings of this study are available on request from the corresponding author.
